# Simulation of the molecular interaction of CRY1A toxins and three Aminopeptidases N from the sugarcane giant borer (*Telchin licus licus*)

**DOI:** 10.1186/1753-6561-8-S4-P107

**Published:** 2014-10-01

**Authors:** Fernando Fonseca, Diogo Martins-de-Sa, Fátima Grossi-de-Sa, Wagner Lucena

**Affiliations:** 1 Embrapa Recursos Genéticos e Biotecnologia, Brasilia, Brazil; 2 Universidade de Brasília, Brasília, Brazil; 3 Embrapa, Brasilia, Brazil

## Background

Accordingly to the pore formation model, *Bacillus thuringiensis *CRY1A toxins, once activated in the midgut of a susceptible insect, participates on a series of binding with protein receptors present in the intestinal epithelium. In *Manduca sexta*, where the mode of action is better characterized, the first interaction consists of a weak binding of a monomeric toxin to the aminopeptidase N (APN) receptor, allowing it´s recognition by Cadherin receptors. The protein-protein interactions induce an oligomer formation of CRY molecules, which is introduced into the plasma membrane, forming a pore that causes osmotic lysis [1]. Although there is plenty information about the activity of CRY1A toxins in *M. sexta*, the same is not observed for *Telchin licus licus*, an insect that is emerging as a major pest of sugarcane fields in Brazil. The present study aimed at simulating and comparing the interaction of CRY1A toxins with APN receptors of *M. sexta *and *T. licus licus *using computational programs.

## Methods

The protein sequence of *M. sexta *APN1 was obtained from the GenBank database [2]. Three APN sequences of *T. licus licus *were isolated from a cDNA library. CRY1Aa and a human APN protein sequences and structures were collected from the PDB database (PDB: 1CIY; 2YD0) while CRY1Ab, CRY1Ac and the insect APNs structures were obtained by homology modeling. Sequence alignment was carried out using the M4T server [3]. Homology modeling was performed using MODELLER 9.10 program. Dynamics simulations were performed using GROMACS 4.5.3. Binding of toxins to the APN receptors were simulated by molecular docking, using the ClusPro metaserver.

## Results and conclusions

In average, 98% of the amino acids in the models were observed in Ramachandran´s allowed regions, indicating that the secondary structures are compatible with the crystallographic data. Root mean square deviation (RMSD) calculated for all the APN and CRY models indicated that the atoms showed the same degree of movement. Root mean square fluctuation (RMSF) calculated for the APN proteins showed that the toxin binding site in *T. licus licus *APNs showed greater flexibility than *M. sexta *APN1[4]. For the CRY toxins, the RMSF showed that loops II and III were more flexible for CRY1Ab. The radius of gyration (Rg) calculated for the APNs indicated that *T. licus licus *APN1 has a greater volume than the other receptors. In the case of the CRY toxins, the Rg was similar between all the proteins. After calculating the solvent accessibility surface (SAS) for all the models, it was possible to observe that the binding site in the toxins as well as the receptors, increases the contact with the water. The molecular docking between the toxins and the receptors indicated that *T. licus licus *APN4 presented closer characteristics of binding when compared with *M. sexta *APN1. These results are the first reports of how the interaction of the toxins and receptors occurs in *T. licus licus´ *organism. The identification of the amino acids that participates in this interaction will be useful for the development of toxins with increased activity for this insect.
